# Resistant Starch Is Actively Fermented by Infant Faecal Microbiota and Increases Microbial Diversity

**DOI:** 10.3390/nu11061345

**Published:** 2019-06-14

**Authors:** Geetha Gopalsamy, Elissa Mortimer, Paul Greenfield, Anthony R. Bird, Graeme P. Young, Claus T. Christophersen

**Affiliations:** 1 Eastern Health Clinical School, Monash University, Box Hill, VIC 3128, Australia; gopa0006@gmail.com; 2 Flinders Centre for Innovation in Cancer, College of Medicine and Public Health, Flinders University, Bedford Park, SA 5042, Australia; elissa.mortimer@flinders.edu.au (E.M.); graeme.young@flinders.edu.au (G.P.Y.); 3 CSIRO Environomics Future Science Platform, North Ryde, NSW 2113, Australia; Paul.Greenfield@csiro.au; 4 CSIRO Health and Biosecurity, Adelaide, SA 5000, Australia; Tony.Bird@csiro.au; 5 School of Medical & Health Sciences, Edith Cowan University, Joondalup, WA 6027, Australia; 6 School of Molecular & Life Sciences, Curtin University, Bentley, WA 6102, Australia

**Keywords:** short-chain fatty acid (SCFA), pH, dietary fibre, gut health, prebiotic

## Abstract

In adults, fermentation of high amylose maize starch (HAMS), a resistant starch (RS), has a prebiotic effect. Were such a capacity to exist in infants, intake of RS might programme the gut microbiota during a critical developmental period. This study aimed to determine if infant faecal inocula possess the capacity to ferment HAMS or acetylated-HAMS (HAMSA) and characterise associated changes to microbial composition. Faecal samples were collected from 17 healthy infants at two timepoints: Preweaning and within 10 weeks of first solids. Fermentation was assessed using in vitro batch fermentation. Following 24 h incubation, pH, short-chain fatty acid (SCFA) production and microbial composition were compared to parallel control incubations. In preweaning infants, there was a significant decrease at 24 h in pH between control and HAMS incubations and a significant increase in the production of total SCFAs, indicating fermentation. Fermentation of HAMS increased further following commencement of solids. Fermentation of RS with weaning faecal inocula increased Shannon’s diversity index (H) and was associated with increased abundance of *Bifidobacterium* and *Bacteroides*. In conclusion, the faecal inocula from infants is capable of RS fermentation, independent of stage of weaning, but introduction of solids increases this fermentation capacity. RS may thus function as a novel infant prebiotic.

## 1. Introduction

The initiation of solid foods in early infant feeding represents a dynamic period of change in the composition of the gut microbiota [1]. This period is also critical to child development with implications for general nutrition and immune development, amongst other benefits [1]. The administration of prebiotics during this period could have profound health consequences [2] as they are non-digestible, generally safe and inexpensive food ingredients that selectively stimulate the growth and/or activity of one or a limited number of bacterial species that already reside in the colon [3].

In adults, high amylose maize starch (HAMS), a cultivar obtained through selective breeding, may function as a prebiotic [4]. It is a form of resistant starch (RS), which is defined as the sum of starch and products of starch degradation which have not been absorbed in the small intestine of healthy individuals and which become available for microbial fermentation in the colon [5]. RS is classified into five types and several of these starches have been shown to alter gut fermentation and change gut microbial composition [5]. HAMS is an example of a type 2 RS. Starches may also be chemically modified (type 4 RS) to reduce their digestibility and obtain favourable physicochemical properties. Acylated type 4 RSs may also increase the delivery of short-chain fatty acids (SCFAs) directly to the colon. One example of this is acetylated-HAMS (HAMSA), which is esterified with acetyl groups.

Due to significant differences in the composition and function of infant and adult gut microbiota, the potential of RS to function as a prebiotic in infants cannot be extrapolated from adult studies. It is possible that the relatively immature gut microbial ecosystem of the infant may not have acquired the necessary diversity of bacteria to ferment a complex carbohydrate such as RS [6,7].

Due to the relative inaccessibility of the proximal colon and portal vein, in vivo measurement of substrate digestion and fermentation requires highly invasive procedures and feeding studies to preweaning infants that are not practical. In vitro static batch fermentation is a rapid, inexpensive method to initially assess substrate fermentation and has been widely used to evaluate the fermentation capacity of both adult and infant faecal inocula [8,9]. Substrate fermentation during in vitro batch fermentation studies is evidenced by substrate disappearance, increased production of SCFAs, a decrease in pH, gas generation, or differences in microbial composition in those ferments containing the substrate when compared to a control. The control incubation does not have an added substrate.

In the present study, we used an in vitro batch fermentation system that simulates human colonic fermentation to determine: (1) The capacity of infant faecal inocula, pre-weaning and weaned, to ferment pre-digested HAMS and HAMSA by monitoring changes in the activity (fermentation) and community structure of the faecal microbiota of preweaning and early weaning infants; and (2) whether the introduction of solids into the diet (weaning) influences these variables.

## 2. Materials and Methods

### 2.1. Study Design

We conducted an observational study in which faecal samples were collected from infants, prior to and following weaning (commencement of first solid foods). The first faecal sample was collected from 8 weeks of age until weaning, while the second sample was collected within 10 weeks of weaning commencing. Collected samples were incubated in vitro with fermentable substrates to address the aims. Each infant was expected to provide two samples, one for each time point. This study complied with National Health and Medical Research Council (Australia) guidelines relating to ethical conduct in human research and was reviewed and approved by the Southern Adelaide Clinical Human Research Ethics Committee (September 2013, 339.13).

### 2.2. Participants and Intervention

Caregivers of preweaning infants were recruited either by direct approach following childbirth and prior to hospital discharge or through advertisements in a local parenting magazine. Inclusion criteria included that the infant was full term at birth with a gestational age of more than 38 weeks; at or above the 10th percentile for weight at birth and with no known cardiac, respiratory or gastrointestinal disease. Infants were excluded if they or their mothers received probiotic supplementation or antibiotics post-delivery and prior to stool collection. The mode of infant feeding, breastfed or bottle-fed or mixed (not exclusively breast fed), was recorded. There were 17 preweaning infants and 16 of these infants went on to provide weaning samples.

### 2.3. Faecal Sample Collection and Processing

Within 15 min of an infant passing a motion, stool was collected from a disposable nappy using a sterile container, placed into an airtight bag and put into a portable freezer set at −20 °C. Within two hours, the sample was processed in the laboratory. Working within an anaerobic cabinet containing 5% H2, 5% CO2, and 90% N2, the faecal samples were homogenised in 50% glycerol (1:1 dilution) and stored at −80 °C until further analysis. The use of frozen samples compared to fresh samples in fermentation experiments has been previously validated [10].

### 2.4. Carbohydrates and Chemicals

The two RSs, HAMS (Hylon VII) and modified HAMS (HAMSA-Crispfilm) were obtained from Ingredion, USA. Hylon VII is composed of 70% amylose and 30% amylopectin. It is estimated that Hylon VII contains 50 g of RS per 100 g [11]. Crispfilm (CF) has a Hylon VII backbone and has undergone a further esterification process to form a starch acetate. The degree of acetylation of CF is less than 2.5%, the limit imposed by U.S. Food and Drug Administration (FDA) food regulation and CF has Generally Recognized as Safe (GRAS) status. Lactulose was obtained from Sigma-Aldrich, Australia.

### 2.5. In Vitro Fermentation

An in vitro pre-digestion step was performed to simulate the digestive action of the infant small intestine prior to the fermentation experiments [12]. See Appendix A.1, for further detail of the pre-digestion method. The in vitro fermentation method was based on the technique described by Edwards et al. [13] and Goni et al. [14]. For each preweaning infant sample there were three groups of incubations: HAMS, lactulose and the control. The control incubation had no additional substrate and lactulose was added to confirm that viable bacteria were present in the faecal inocula of each subject. For weaning infant samples there were four incubations, with HAMSA as the additional group, along with HAMS, lactulose and the control. HAMSA was not tested in preweaning infants due to small sample volumes. All fermentations were performed in triplicate for each donor/substrate at 0 and 24 h time points. Briefly, 100 mg of pre-digested starch residue was weighed into triplicate 15 mL sterile culture tubes together with 8–10 sterile 2.5 mm glass beads and 9 mL of autoclaved fermentation media. Frozen homogenised faecal material from participants was thawed and a 10% *w*/*v* faecal slurry was prepared by homogenisation and dilution in pre-reduced phosphate-buffered saline (PBS) (0.1 M, pH 7.2). Working within an anaerobic chamber, 1 mL of faecal slurry was added to each fermentation tube (1% *w*/*v*). Controls were incubated in parallel with incubations containing HAMS, lactulose or HAMSA. Tubes were incubated under anaerobic conditions with gentle agitation for 24 h. Fermentation was terminated at 0 h (Blanks) and at 24 h samples by centrifugation at 13,000× *g*, 4 °C for 10 min and the supernatant was stored at −80 °C for further measurements.

### 2.6. SCFA and pH

SCFA concentration was determined using capillary column gas chromatography according to McOrist et al. [15]. A digital pH meter was used to measure pH. Fermentation of the substrate was deemed to have occurred if there was a statistically significant decrease in pH and an increase in production of SCFA at 24 h in ferments containing added RS when compared to the respective control.

### 2.7. Molecular and Sequence Analysis

#### 2.7.1. DNA Extraction

DNA was extracted from ferments using bead beating followed by the MoBio PowerMag Microbiome RNA/DNA isolation kit (Qiagen, Hilden, Germany) optimised for epMotion (Eppendorf, Hamburg, Germany) platforms (see Appendix A.2). Due to the recognised variation in fermentation between replicates during in vitro batch fermentation [16], DNA was extracted from a pooled sample containing 1 mL from each ferment replicate.

#### 2.7.2. Real-time Quantitative Polymerase Chain Reaction (qPCR)

Selected bacteria including *bifidobacterium*, *lactobacillus* and total bacteria, were quantified by specific primers targeting the 16S rRNA gene using qPCR. See Appendix A, Table A1, for primer sequences and the optimised qPCR conditions. The ability of a substrate to selectively stimulate the growth of a given bacterial taxon was determined by comparing incubations with either HAMS or HAMSA to the 24 h control. All qPCR analysis was performed on the CFX 384TM real-time PCR detection system (Bio-Rad, Hercules, CA, USA) (See Appendix A.3). Absolute abundance was estimated according to Christophersen et al. [17].

#### 2.7.3. Sequencing of 16S Ribosomal RNA Encoding Gene Amplicons

16S ribosomal DNA gene sequencing was performed on DNA extracted from each participant’s 24 h fermentation samples (preweaning control, preweaning HAMS, weaning HAMS, weaning HAMSA, weaning control). The 24 h samples were chosen as the fermentation is not only affected by the added substrate but also by the remaining substrates in the faecal slurry. We therefore believe the true control for each subject and substrate is a control fermentation with no added substrate to take into account the available substrate in the faecal slurry. The methods outlined in Illumina’s 16S Metagenomic Sequencing Library Preparation protocol (Illumina, San Diego, CA, USA) were followed with minor adjustments made to PCR thermal cycle conditions, as described in Appendix A.4.

#### 2.7.4. Taxonomic Assignments to 16S Reads

An in-house (CSIRO) amplicon clustering and classification pipeline (GHAP) based on tools from Usearch [18] and a Ribosomal Database Project (RDP) classifier [19] combined with locally written tools for demultiplexing and generating Operational Taxonomic Unit (OTU) tables were used to process the amplicon sequence data. Following the merging of paired reads, dereplication, clustering at 97% and chimera checking were also performed using the pipeline. Classification of the reads was then performed by using the RDP to assign taxonomy and by finding the closest match to the OTU from a set of reference 16S sequences [19]. OTUs were defined at a 97% sequence similarity level and classified to genus level. Sequences which were not classified using the pipeline were manually blasted against the NCBI database.

### 2.8. Statistical Analysis

For SCFA and pH results, data normality was assessed using the Shapiro–Wilk test using SPSS Version 22.0. A boxplot of the dataset was used to identify outliers within preweaning and weaning groups, Univariate ANOVA with Bonferroni correction was used to analyse for differences in starting pH and total SCFA of the different groups within the weaning and preweaning infants. Due to differences in the number of formula and breastfed infants, a general linear mixed model was used to determine if within the preweaning group, the method of feeding influenced the effect of incubation with HAMS on both change in pH and total SCFA production. A repeated measures two-factor ANOVA was used to determine if there was an effect of weaning on parameters of HAMS fermentation (pH and total SCFA) when compared to controls. Values are presented as means ± their standard errors. Statistical significance was accepted as *p* < 0.05.

For analysis of the molecular results, the qPCR values were log10 transformed and the means were compared using Student’s *t*-test. Microbial abundance at 24 h was compared with those at 0 h for each substrate. Multivariate analysis of the sequencing data was performed using PRIMER 7 with PERMANOVA (PRIMER-e, Auckland, New Zealand). Statistical analysis of Bray-Curtis dissimilarities were calculated using relative abundance of bacterial genera at the family level following 24 h of fermentation. Alpha diversity index was also calculated at the family level. Principal coordinate analysis (PCOA) was used to visualise the dissimilarity data.

## 3. Results

The seventeen participants provided a preweaning sample, with all but one of these infants also providing a second (weaning) sample (see Table 1).

### 3.1. SCFA and pH Levels

The Shapiro–Wilk test confirmed a normal distribution of faecal pH and SCFA data in both preweaning and weaning incubation samples. One participant, in the preweaning exclusively breast-fed group, was noted to have an outlier for total SCFA for each substrate. Parallel series of calculations performed with and without the inclusion of this participant’s data did not alter the final statistical conclusions. Within the preweaning group, linear mixed model analysis revealed no effect of mode of feeding (exclusively breast fed or mixed) on SCFA (*p* = 0.754) or pH (*p* = 0.809), following incubation with either substrate (HAMS or lactulose).

Results for initial, final and change in pH at 24 h following incubation are presented in Table 2. All incubations, including the lactulose, resulted in a significant decrease in pH at 24 h. In both weaning and preweaning groups the decrease in pH was significantly greater following incubation with HAMS than in the controls, consistent with active fermentation of HAMS. It was expected to observe a decrease in pH for control samples due to residual substrate in the faecal inoculate. In the weaning group, incubation with HAMSA also led to a decrease in pH when compared to the control.

Analysis of variance showed no effect of the substrates on initial pH, *p* = 0.36. A repeated measures two-factor ANOVA revealed an effect of HAMS *p* < 0.001, but not stage of weaning on change in pH, *p* = 0.34. There was no interaction between the substrate and stage of weaning *p* = 0.54, indicating that the introduction of solids did not influence the effect of incubation with HAMS on change in pH when compared to controls. The means and standard error thereof for total and major individual production of SCFAs at 24 h are shown in Table 3. In the faecal inocula of the preweaning group there was a significant increase in the concentration of SCFAs, acetate and butyrate when HAMS was added compared to the controls, indicating that the preweaning faecal inocula had some capacity to utilise HAMS as a substrate.

In the faecal inocula from weaned infants, there was a significant increase in the production of total SCFAs, acetate and propionate for both HAMSA and HAMS. A significant increase in butyrate was seen only with HAMS and not HAMSA. These changes confirmed fermentation of the RSs by the infant faecal inocula. The molar ratios for all the substrates confirmed previous findings that, in young infants, acetate is by far the dominant SCFA during fermentation.

In relation to total SCFA concentration, a two-way repeated measures ANOVA demonstrated a significant interaction between incubation with HAMS and stage of weaning, *p* = 0.015, suggesting that the fermentation of HAMS is enhanced post the introduction of solids. A pair-wise comparison with Bonferroni correction revealed no statistically significant effect of weaning on total SCFA concentration, *p* = 0.06. However, there was a statistically significant effect for HAMS *p* < 0.001.

### 3.2. Microbial Community Analysis

Across all samples collected at 24 h there were 321 operational taxonomic units identified, with an average of 94 OTUs per sample (59–213). For preweaning faecal fermentation samples, a total of 632,032 usable reads were obtained and for weaning, 873,706 reads were obtained for downstream analysis. DNA from two samples from the preweaning group failed to amplify during library preparation for unknown reasons and therefore these participants were omitted from sequence analysis. Sequences were classified at each phylogenetic level from phylum to genus.

#### 3.2.1. Alpha Diversity

**Figure 1:**
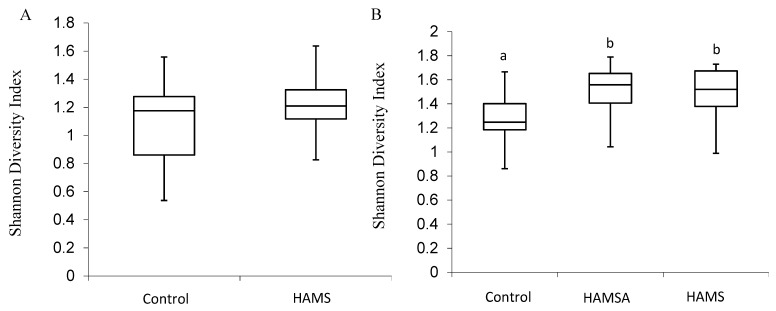
Boxplot of diversity at family level in (**A**) preweaning and (**B**) weaning infant faecal inocula following 24 h in vitro fermentation. Boxes indicate 25th to 75th percentiles, with mean values marked as a line and whiskers indicating minimum and maximum values. Different letters mean significantly different from each other (*p* < 0.05).

Alpha diversity was calculated in this study, although the study uses an in vitro model (closed system), because changes in diversity can still be observed as the Shannon index combines species richness and their relative abundances. In the preweaning group at 24 h of incubation, there was no significant difference in the Shannon Index between bacterial communities following incubation with HAMS or the controls. For example, at the family level, a one-way ANOVA determined that, in the preweaning group, the overall mean of the log(e) of the Shannon Index did not differ between the control and the HAMS incubations (*p* = 0.10). The Shannon Diversity Index boxplot based on OTU abundance at the family level is presented in Figure 1A. In the weaning group, at the family level, there was a significant increase in the Shannon index in the RS groups compared to the controls (*p* = 0.05). However, the type of RS (HAMSA or HAMS) did not have an effect on this measure of diversity. The Shannon Diversity Index boxplot based on OTU abundance at the family level for the weaning infants is presented in Figure 1B.

#### 3.2.2. Beta Diversity

**Figure 2:**
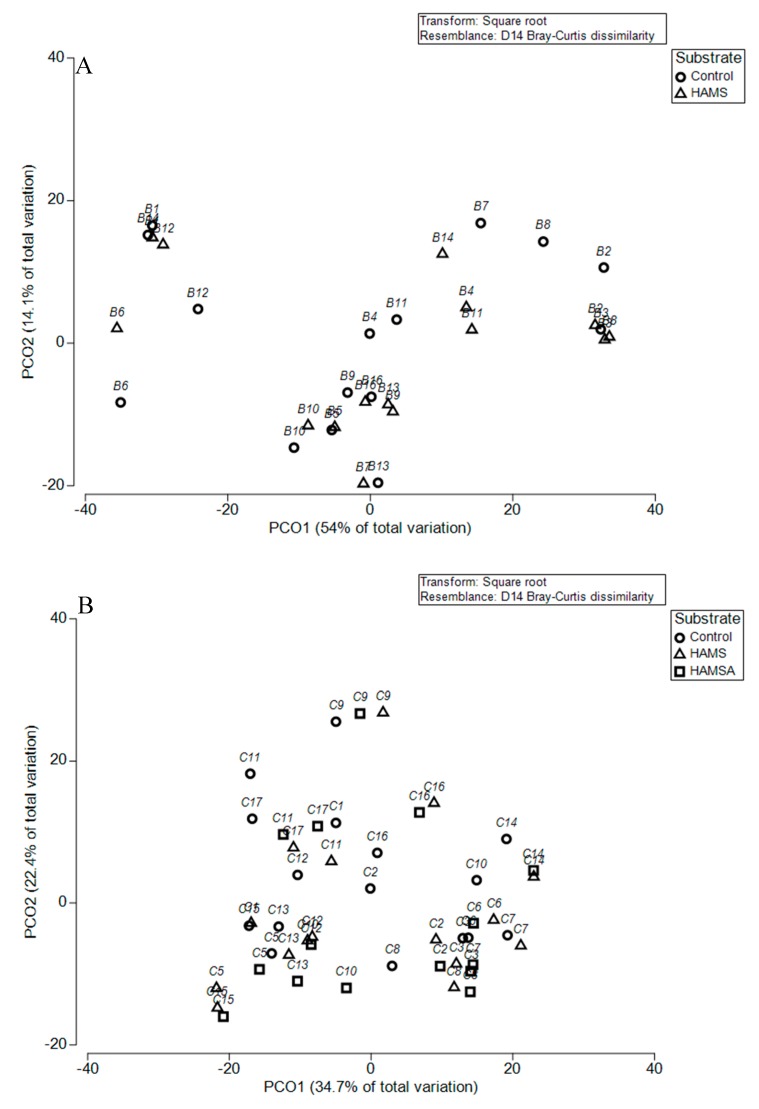
Principal coordinate analysis of the Bray-Curtis dissimilarity matrix for (**A**) preweaning and (**B**) weaning samples, calculated at the family level. PCO: Principal Coordinate Analysis.

Among the preweaning samples, multivariate analysis did not reveal an effect of HAMS on microbial community structure (*p* > 0.05). However, in the weaning samples, the effect of both HAMS and HAMSA were significant (*p* < 0.05), compared to the controls. Pairwise comparison was performed to investigate the differences between the two test groups. This demonstrated that both RSs had a similar effect on community structure (*p* = 0.97) (see Figure 2). It is apparent that samples from the same individual clustered closely.

#### 3.2.3. Relative Abundance

In order to identify the changes in the composition of bacterial communities that might utilise the RSs, the relative abundance of bacterial groups in fermentation fluid at 24 h of incubation was assessed. The relative abundance was calculated and is presented in Appendix A, Table A2. Across all levels of classification, there were a number of statistically significant differences in the relative abundance of bacteria between HAMS and controls in the weaning group when compared to the preweaning group. At the phyla level in weaning infants, there was a significant increase in the proportion of Actinobacteria and Bacteroidetes at 24 h of fermentation for both RSs in comparison to the controls (*p* < 0.05). There was also a significant reduction in Proteobacteria following incubation with both HAMS and HAMSA (*p* < 0.05). At a genus level in weaning infants, the abundances of *Bacteroides* and *Bifidobacterium* were significantly increased following incubation with the RSs when compared to controls (*p* < 0.05).

**Figure 3:**
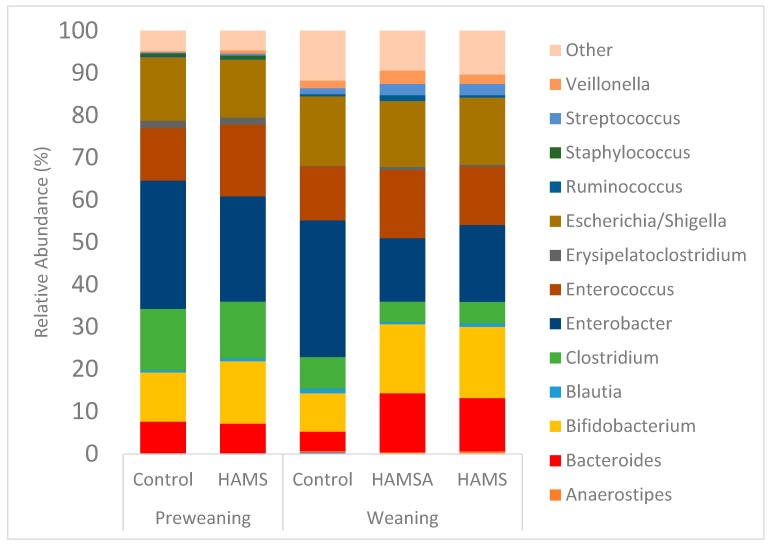
Genus-level composition of the microbial community after sequencing. DNA from 24 h in vitro fermentations of infant faecal inocula. Bacterial genus with a relative abundance of less than 1% are grouped as “other”.

Incubation with both HAMS and HAMSA led to a concomitant reduction in the relative abundance of *Enterobacter*. Compared to the control, the relative abundance of *Ruminococcus* was significantly increased following incubation with HAMSA (*p* = 0.01), but not HAMS (*p* = 0.58). Figure 3 illustrates microbial composition after 24 h of fermentation at a genus level for each substrate, with a relative abundance threshold of 0.1%.

#### 3.2.4. Quantitative PCR

All qPCR assays had previously been verified using single cell colony sequencing and they were found to be 100% specific to the bacterial group assigned. In preweaning infants, the absolute abundance for total bacteria increased after 24 h of fermentation in HAMS and controls (see Table 4) compared to time 0 h in the controls (representative of the baseline). However, at 24 h the absolute abundance of total bacteria, bifidobacteria and *Lactobacillus* did not differ between the control and the HAMS incubations. In the weaning infants, following 24 h of in vitro fermentation, the absolute abundance of total bacteria, bifidobacteria and *Lactobacillus* increased in all three groups (control, HAMS and HAMSA) compared to time 0 h controls. While there was no difference in the absolute abundance of total bacteria or *Lactobacillus* at 24 h compared to the control, the absolute abundance of *Bifidobacterium* was significantly greater in HAMS and HAMSA compared to controls. This indicates that both RS’s can stimulate the growth of *Bifidobacterium* in the faecal microbiota of weaning infants.

## 4. Discussion

A divergence in the intake of fibre between those living in Western countries and those consuming a predominantly agrarian diet, as occurs in many rural parts of the world, emerges as soon as solids are introduced into the infant diet [1]. Thus, targeted manipulation of fibre content in the early diet may affect the emerging gut microbiota during a critical period and lead to functional and compositional changes which could benefit the host’s developing immune system [20,21]. It is within such a context that we were interested in whether forms of RS, which function as a prebiotic in adults, could have a role in infancy.

In both the weaning and preweaning groups, the production of SCFAs by the faecal inocula from these infants was significantly greater in the presence of HAMS when compared to controls. While fermentation might be expected in faeces from the weaning cohort, it is surprising that the microbiota of preweaning infants already possess capacity to ferment starch. However, this capacity did increase significantly post commencement of complementary feeds.

Previous results regarding the potential of young infant faecal bacteria to ferment complex carbohydrates, are conflicting. An early study by Parrett et al. suggested that the capacity of human faecal microbiota to ferment complex carbohydrates does not emerge for several months after solids are commenced [6]. In contrast, Christian et al. found that the faecal microbiota of early weaning infants is highly efficient at fermenting the digestible waxy maize starch [8]. Heterogeneity in methodologies across in vitro fermentation studies, including the inclusion or exclusion of substrate pre-digestion, the age of study participants and the preparation of faecal inocula, limits comparison of results across studies. In this study, we used a substrate pre-digestion which reflects the low level of alpha-amylase in the infant gut.

There was no significant difference in the production of total SCFAs, especially acetate, between HAMS and HAMSA (which is acetylated HAMS). It might be that the degree of substitution was insufficient for cleavage of the esterified acetate to achieve a statistically significant increase in the overall pool of acetate. It could also be that infant microbiota might not have yet acquired the esterase capacity needed to cleave the additional acetate.

Surprisingly, and despite the fermentation findings, our molecular findings showed little difference in bacterial composition following incubation of HAMS with the preweaning infant inocula in comparison to the controls. There were no selective differences in the abundance of groups of bacteria following incubation of HAMS with the preweaning infant inocula, in comparison to the controls. However, within ten weeks of commencing solids, the molecular changes within the weaning infant faecal incubations suggest emergence of such a capacity. If selective stimulation of *Bifidobacterium* and an increase in the production of SCFAs are components of the prebiotic definition [2] then, based on these findings, HAMS and HAMSA may well function as a novel prebiotics during infancy.

A significant number of *Bifidobacterium* spp. possess genes belonging to the GH13 family of glycosyl hydrolases (GH) [22,23]. Enzymes belonging to this family are heavily involved in the degradation of starch and starch related-substrates [24]. Despite the relatively high abundance of *Bifidobacterium* in preweaning infant inocula, several possibilities exist for why there were no significant differences in microbial profile between controls and substrate incubations. It could be that the significant inter-individual variation in the gut microbial composition of preweaning infants might have masked the capacity to establish substrate level differences in the microbial profile [25]. Another explanation rests upon differences in metabolic function amongst various members of the *Bifidobacterium* genus. Depending on the particular ecological niche, given the myriad of metabolic pathways available to *Bifidobacterium* spp., selective pressure will support the growth of those *Bifidobacterium spp.* that are able to utilise the substrates that are most available [22]. Metagenomic studies have demonstrated differences in which bifidobacterial GH genes are transcribed between infants and adults [26]. For example, in breast feeding infants, there is greater transcription of bifidobacterial GH genes that are involved in the degradation of Human Milk Oligosaccharides (HMO) and mucin, compared to adults which reveals a greater predominance of bifidobacterial GH encoding genes involved in the breakdown of complex plant-derived carbohydrates [26]. Following the introduction of solids, when more complex carbohydrates escape the digestive enzymes of the host and are encountered by the infant gut microbiota, those microbial genes that are involved in the utilisation of these novel nutrients will be switched on [27]. Given the immaturity of pancreatic function in the young infant, following the initiation of solids, significant amounts of dietary starch will enter the infant’s colon. This will favour a selective increase in the expression of genes associated with starch utilisation and may account for the findings in the weaning faecal inocula.

Despite the recognised relevance of *Bifidobacterium* to starch utilisation, it had been suggested that *Ruminococcus bromii,* a member of the Firmicutes, is a keystone species in the degradation of HAMS [28]. Our results cannot confirm this assertion. While there was a selective increase in the relative abundance of *Ruminococcus* spp. following incubation with HAMSA, there was no such increase following incubation of HAMS with the weaning infant inocula. This was despite evidence of starch utilization, as seen in the increase in the relative abundance of other groups of bacteria in comparison to the controls. This suggests that many strains of bacteria are able to utilise HAMS, which aligns with studies conducted in pigs [29,30]. Members of Bacteroidetes, a dominant bacterial phylum in the mammalian gut, also encode numerous discrete polysaccharide utilisation loci (PULs) that may facilitate starch utilisation. Incubation of weaning infant faecal inocula with both RSs led to an increase in the relative abundance of Bacteroidetes and an increase in the ratio of Bacteroidetes:Firmicutes. An increase in the ratio of Bacteroidetes:Firmicutes may be associated with several positive health outcomes, including reduced risks of obesity and intestinal inflammation [31].

The concordance between HAMS and HAMSA in relation to the effects on microbial composition and diversity observed in this study could be due to the minimal nature of the chemical modification. The HAMSA starch used in this study is approved for consumption in infant foods. For starch acetate, the FDA and the WHO have stipulated an acetyl group’s percentage below 2.5 g/100 g (corresponding to a maximum Degree of Substitution (DS) of 0.1) for a food application. Although HAMS with higher degrees of acetylation have been shown to facilitate increased delivery of SCFA into the human colon, their comparable effects on the gut microbiota have not yet been examined [32].

In a study feeding modified and unmodified RS to adult participants, different effects on faecal microbiota composition between the two starches were found [33]. The authors found the intake of the modified, but not the unmodified starch, over a three-week period led to a significant increase in the abundance of Actinobacteria and *Bacteroides*. Using our in vitro model, we demonstrated a similar taxonomic effect following incubation of the weaning infant feces with the modified RS. However, in contrast to Martinez et al. (2010) we found incubation of the unmodified RS led to similar taxonomic changes at the phylum level. From a translational viewpoint, if HAMS is eventually used in infant nutrition, the unmodified form may have greater appeal to caregivers due to its lack of chemical modification.

Being an in vitro design, our study has inherent limitations. The high relative abundance of Proteobacteria at 24 h of incubation, particularly in the controls, reflects a difference between in vitro and in vivo conditions and suggests that in vitro conditions might have favoured the growth of Proteobacteria. This also reflects that not all bacteria grow in vitro and faeces contain a large proportion of dead bacteria which may not be representative of the living mucosal-adherent and/or luminal bacteria in the gut. The limited availability of infant faecal inocula also precluded comparative evaluation of the fermentation of the chosen RS to other substrates which may already be recognised as prebiotics in infants, e.g., fructo-oligosaccharides (FOS). To circumvent this, it might have been possible to pool the faecal samples from different donors. However, individual variation in the microbiota might be greater than the effects of treatment.

A further limitation was that the actual degradation of the substrate in each ferment was not measured. It is not uncommon for in vitro fermentation studies to omit this measurement, instead relying, as we have done, on the products of fermentation and changes in microbial composition to provide an estimate of fermentation. It should also be noted that, irrespective of the benefits to gut health, RS may impact the nutritional status of the infant diet. Therefore, further studies are required to determine a safe dose of RS for highly vulnerable populations, such as growing babies and children.

Ultimately, any claim to prebiotic status for HAMS during the infant period must be supported by well-designed human studies. Inulin, fructo-oligosaccharides and galacto-oligosaccharides, the subjects of several randomised controlled trials, are generally held to be the main substrates to have prebiotic potential during infancy. However, these agents are costly to produce and, particularly in the case of FOS and galacto-oligosaccharides (GOS) due to their small size, confer a luminal osmotic effect and are rapidly fermented. These features carry the risk of precipitating undesirable clinical effects, such as diarrhoea and abdominal discomfort. HAMS has several advantages. It can be readily cultivated, is slowly fermented and does not produce an osmotic effect in the large intestine. It can also be readily incorporated into foods without altering the processing or organoleptic properties [34].

## 5. Conclusions

This study confirms that faecal inocula, whether preweaning or at weaning infants has the capacity to utilise HAMS and HAMSA as a potential substrate. Incubation with both starches selectively stimulated *Bifidobacterium* copy numbers and increased the Bacteroidetes:Firmicutes ratio, outcomes that, if replicated in direct feeding studies, may be associated with beneficial health outcomes. These findings justify further in vivo infant studies to examine the short- and long-term effects of different doses of HAMS during weaning on the composition and function of the emerging gut microbiota and clinical outcomes.

## Figures and Tables

**Table 1:** Description of infants used as faecal donors for in vitro fermentation experiments.

Participants	Preweaning (*n*)	Weaning (*n*)	Age Preweaning Sample (Months) Mean ± SE	Age Weaning Sample (Months) Mean ± SE
Exclusively Breast-Fed	10	10	3.32 ± 0.37 ^a^	7.03 ± 0.27 ^b^
Mixed	7	6	3.29 ± 0.22 ^a^	7.39 ± 0.21 ^b^
Total	17	16	3.31 ± 0.23	7.16 ± 0.18

SE: Standard error. Means in a row without a common superscript letter differ significantly (*p* < 0.05).

**Table 2:** Initial and final change in pH following incubation of infant faecal microbiota with high amylose maize starch (HAMS) and other substrates. Mean ± SE.

	Preweaning			Weaning			
	Control	HAMS	Lactulose	Control	HAMS	HAMSA	Lactulose
Initial pH (0 h)	7.64 ± 0.03 ^a^	7.63 ± 0.03 ^a^	7.69 ± 0.03 ^a^	7.66 ± 0.03 ^a^	7.65 ± 0.02 ^a^	7.65 ± 0.03 ^a^	7.72 ± 0.04 ^a^
Final pH (24 h)	6.72 ± 0.08 ^a^	6.40 ± 0.09 ^b,^*	4.73 ± 0.12 ^c,^*	6.77 ± 0.05 ^a^	6.43 ± 0.05 ^b,^*	6.37 ± 0.04 ^b,^*	4.65 ± 0.08 ^c,^*
Δ pH	−0.93 ± 0.07 ^a^	−1.21 ± 0.06 ^b,^*	−3.03 ± 0.06 ^c,^*	−0.82 ± 0.07 ^a^	−1.14 ± 0.08 ^b,^*	−1.09 ± 0.10 ^b,^*	−2.89 ± 0.20 ^c,^*

Within the preweaning and weaning groups a one-way ANOVA was used. Unlike superscript letters within each row are significantly different (Bonferroni adjusted *p* < 0.05). * defined as different from control (*p* < 0.05). HAMSA: High Amylose Maize Starch Acetylated.

**Table 3:** Short-chain fatty acid (SCFA) concentrations (mmol/L) following incubation with test substrates using pre- and weaning infant faecal inoculum. Mean ± SE.

	Preweaning			Weaning			
	Control	HAMS	Lactulose	Control	HAMS	HAMSA	Lactulose
Total SCFA ^#^	16.68 ± 1.7 ^a^	23.70 ± 1.7 ^b,^*	61.84 ± 5.4 ^c,^*	20.11 ± 2.3 ^a^	34.85 ± 4.0 ^b,^*	37.81 ± 4.2 ^b,^*	78.27 ± 4.5 ^c,^*
Acetate	14.70 ± 1.5 ^a^	20.93 ± 1.5 ^b,^*	56.11 ± 6.5 ^c,^*	16.98 ± 2.1 ^a^	27.73 ± 3.3 ^b,^*	30.27 ± 3.1 ^b,^*	73.42 ± 5.0 ^c,^*
Propionate	0.83 ± 0.17 ^a^	1.33 ± 0.33 ^a^	1.13 ± 0.80 ^a^	1.60 ± 0.32 ^a^	4.14 ± 0.91 ^b*^	5.56 ± 1.2 ^c*^	2.89 ± 0.74 ^a,b,c,^*
Butyrate	0.36 ± 0.13 ^a^	0.74 ± 0.26 ^b^	0.27 ± 0.12 ^a^	1.09 ± 0.26 ^a^	2.20 ± 0.59 ^b*^	1.23 ± 0.33 ^a^	1.81 ± 1.1 ^a^

^#^ Total SCFA (mmol/L) = sum of acetate, propionate, butyrate and minor SCFAs (valeric, caproic, isobutyric, isovaleric). Within the preweaning and weaning groups a one-way ANOVA was used. Unlike superscript letters within each row are significantly different (Bonferroni adjusted *p* < 0.05). * defined as different from control (*p* < 0.05).

**Table 4:** 16S rDNA copy numbers (log 10 copy numbers mL^−1^ fermentation effluent) of specific bacterial groups before (0 h) and after 24 h of in vitro fermentation with infant faecal inocula and test substrates, as determined by qPCR. Mean ± SE.

		Total Bacteria		*Lactobacillus*		*Bifidobacterium*	
	Substrate	0 h	24 h	0 h	24 h	0 h	24 h
Preweaning	control (*n* = 17)	6.6 ± 0.15	7.18 ± 0.24 ^a,^*	4.26 ± 0.4	4.19 ± 0.23 ^a^	6.06 ± 0.09	6.08 ± 0.32 ^a^
	HAMS (*n* = 17)		7.21 ± 0.1 ^a,^*		4.02 ± 0.28 ^a^		6.39 ± 0.25 ^a^
Weaning	Control (*n* = 16)	6.65 ± 0.07	7.32 ± 0.06 ^a,^*	2.9 ± 0.27	3.34 ± 0.86 ^a^	5.09 ± 0.31	5.56 ± 0.21 ^a^
	HAMSA (*n* = 16)		7.42 ± 0.06 ^a,^*		3.52 ± 0.91 ^a^		6.21 ± 0.10 ^b,^*
	HAMS (*n* = 15)		7.47 ± 0.05 ^a,^*		3.49 ± 0.89 ^a^		6.04 ± 0.19 ^b,^*

In the preweaning and weaning groups, within column values which do not share a superscript letter are significantly different (*p* < 0.05). * Significant difference between 0 and 24 h values for each substrate, *p* < 0.05.

## Appendix A

### Appendix A.1. Pre-Digestion Method

5 g of test starch was accurately weighted into tubes (8) and incubated with 25 mL of pepsin (1 g/mL, made up in 0.02 mol HCl/L, pH 2, for 30 min at 37 °C. The next phase of digestion was replicated by neutralisation with sodium hydroxide (0.02 M) and incubation with 25 mL of amyloglucosidase (200 U/mL) and alpha-amylase (1.5 U/mL) solution for 5 h in a shaking water bath at 37 °C. Following the incubation, contents were pooled and transferred into a beaker containing 80% ethanol and left overnight. The supernatant was then removed and the residue was washed with 80% ethanol. This was then centrifuged (2000× *g* for 10 min), followed by the removal of the supernatant. Washing with alcohol, centrifuging and removal of the supernatant was repeated four times. Following air-drying in the fume hood for another 24 h, the residue was collected for use in the small-scale batch in vitro fermentation method. Only one batch of pre-digestion was performed for each substrate and this provided sufficient amounts for all the required incubations.

### Appendix A.2. DNA Extraction Method

DNA was extracted using the PowerMag Microbiome DNA Isolation kit (Qiagen, Hilden, Germany) from a pooled sample containing 1 mL from each ferment replicate. No DNA was extracted from ferments incubated with lactulose as human faecal microbiota of all age groups are recognised to readily ferment lactulose. The purpose of incubation with the lactulose was only to confirm the viability of the faecal microbiota from the particular participant. A sample of 1 mL from each of triplicate ferments was transferred into three separate 2 mL Eppendorf tubes and centrifuged at 13,000× *g* at 4 °C for 5 min using a bench centrifuge (Eppendorf, Hamburg, Germany). Each resulting cell pellet was retained and the supernatant discarded. Heated PowerMag Microbiome lysis solution (650 μL) was added to the first of the three tubes (each containing the cell pellet) and mixed thoroughly. All the solution was then transferred into the second tube, mixed, and the contents transferred into the third tube, which was also subjected to vigorous mixing. Sterile 0.4 g PowerMag glass beads were added to the final tube and the sample was homogenised for 3 min at maximum speed on a Mini-Beadbeater (BioSpec Products, Bartlesville, OK, USA). The tube was then centrifuged for 5 min at 13,000× *g* at room temperature. The supernatant was transferred into fresh PCR-grade 1.75 mL tubes. 30 μL of Proteinase K was added to the supernatant, mixed and kept for 10 min at 70 °C. 30 μL of PowerMag Inhibitor Removal Solution was then added to each sample and mixed well. The samples were incubated at room temperature for 5 min and then were again centrifuged at 13,000× *g* and the supernatants transferred to a fresh 2 mL Deep Well Plate, ensuring no transfer of any residual pellet. 5 μL of RNAse was added to each well of this Deep Well Plate.

The extraction method was completed on the Eppendorf epMotion 5075TMX platform following the manufactures instructions using the PowerMag Microbiome DNA Isolation kit. Reagents used in this programme included Clear Mag Wash Solution, the Clear Mag Binding Solution and Clear Mag Beads. Briefly, using the automated wash and elution protocol, beads and a binding solution were added to each well and mixed together. In this process, the DNA would adhere to the beads after which the plate was transported to a magnet and the waste removed. This process was repeated 3 times with the lysate and binding solution followed by an ethanol wash. Low heat was applied to remove traces of ethanol and an elution buffer was added. This released the nucleic acids from the beads and the elute was separated from the beads and stored at −80 °C.

**Table A1:** PCR primers and their amplification conditions.

Target	Primers	Sequence (5′–3′)	Conc (nM)	Annealing		References
				Temp (°C)	Time (s)	
Total Bacteria	UnivF	TCCTACGGGAGGCAGCAGT	500	60	45	[35]
	UnivR	GGACTACCAGGGTATCTATCCTGTT				
Lactobacillus Spp	Lacto-F	AGCAGTAGGGAATCTTCCA	500	56	20	[36]
	Lacto-R	CACCGCTACACATGGAG				
Bifidobacterium Spp	Bifi-F	TCGCGTCCGGTGTGAAAG	500	56	20	[37]
	Bifi-R	CCACATCCAGCGTCCAC				

### Appendix A.3. qPCR

All qPCR analysis was performed on the CFX 384TM real-time PCR detection system (Bio-Rad, Hercules). Reactions were performed in triplicate, with a total reaction volume of 10 µL. Each reaction consisted of 3 µL (2.8 ng/μL) of DNA template and 7 µL PCR mixture containing 5µL of SYBR mix, bovine serum albumin (0.2 µL), forward (0.1 µL) and reverse primers (0.1 µL) (2.5 ng/µL) and PCR-grade water (1.6 µL). The qPCR cycling conditions had an initial hot start at 98 °C for 3 min, followed by 35 cycles of two step qPCR with denaturing at 98 °C for 15 s using the annealing/elongation temperatures as in Table 1. Fluorescence intensities were detected during the last step of each cycle. qPCR melting curves were obtained after amplification by continuously collecting fluorescence intensity measurements as the reactions were slowly heated from 55 to 95 °C in increments of 0.50 °C/s.

### Appendix A.4. Next Generation Sequencing

The methods outlined in Illumina’s “16S Metagenomic Sequencing Library Preparation” protocol were followed [38] with adjustments made to PCR thermal cycle conditions, as detailed below. The hypervariable region V4 of the 16SrRNA gene was amplified from the extracted DNA using modified primer pairs with Illumina adapter overhang sequences. The full-length primer sequences using standard IUPAC nucleotide nomenclature were:

16S Amplicon PCR Forward Primer =
  5′-TCGTCGGCAGCGTCAGATGTGTATAAGAGACAGGTGCCAGCMGCCGCGGTAA-3′
Amplicon PCR Reverse Primer =
  5′-TCTCGTGGGCTCGGAGATGTGTATAAGAGACAGGGACTACHVGGGTWTCTAAT-3′

A two-step PCR process was required. The PCR reaction contained 5 µL of forward primer (1 nM), 5 µL of reverse primer (1 nM) and 12.5 µL of 2 × KAPA H-iFi Hotstart ReadyMix (KAPA Biosystems, Wilmington, MA, USA) in a total volume of 25 µL. The PCR reaction was performed on a Veriti Thermal Cycler (Thermo Fisher Scientific, Waltham, MA, USA) using the following programme: 25 cycles of 95 °C for 30 s, 55 °C for 30 s, 72 °C for 30 s followed by holding at 72 °C for a further 5 min. Following a clean-up of the PCR product, indexing PCR was performed. This step used the Nextera XT Index Kit to attach the dual indices and the Illumina sequencing adapters. The manufacturer’s instructions were followed as described in the Illumina library preparation protocol, as mentioned above. The final library was pair-end sequenced using a MiSeq Reagent Kit v3 on the Illumina MiSeq platform. Library preparation and sequencing was performed at Flinders University, South Australia. A standard *t*-test was used to compare the relative abundances of control and test groups at the different taxonomic levels (see Table A2).

**Table A2:** Percent abundance of the dominant major bacterial taxa at each taxonomic level.

Taxonomy	Preweaning (*n* = 15)			Weaning (*n* = 14)				
	Control	HAMS	*p* Value	Control	HAMS	*p* Value	mHAMS	*p* Value
**Phylum**								
Actinobacteria	12.50	15.48	0.35	10.16	18.55	0.004	17.88	0.004
Bacteroidetes	7.87	7.29	0.86	4.73	13.04	0.001	15.56	0.03
Firmicutes	33.33	37.74	0.09	33.5	32.51	0.79	34.37	0.11
Proteobacteria	46.23	39.39	0.17	51.27	35.76	0.001	32.06	0.004
**Class/subclass**								
Actinobacteria	12.51	15.48	0.35	10.17	18.56	0.004	17.88	0.004
Bacilli	13.46	18.74	0.02	14.48	16.83	0.32	19.04	0.08
Bacteroidia	7.87	7.29	0.86	4.73	13.04	0.001	15.56	0.001
Clostridia	17.71	16.28	0.58	16.25	12.31	0.26	10.79	0.07
Gammaproteobacteria	46.10	39.13	0.17	50.69	35.19	<0.001	31.56	<0.001
**Order**								
Bacteroidales	7.87	7.29	0.86	4.73	13.04	0.001	15.56	0.001
Bifidobacteriales	11.64	14.73	0.32	9.12	16.83	0.01	16.33	0.01
Clostridiales	17.71	16.27	0.58	16.24	12.30	0.26	10.79	0.07
Enterobacteriales	45.98	39.04	0.17	50.61	35.16	<0.001	31.51	<0.001
Erysipelotrichales	1.77	1.73	0.93	0.25	0.54	0.07	0.77	0.09
Lactobacillales	12.81	17.67	0.03	14.44	16.58	0.36	19.03	0.08
**Family**								
Bifidobacteriaceae	11.67	14.82	0.32	9.31	17.54	0.007	16.86	0.004
Bacteroidaceae	7.49	7.06	0.89	4.60	12.91	<0.001	14.34	0.002
Enterococcaceae	12.42	17.04	0.03	12.79	13.96	0.61	16.32	0.16
Clostridiceae	14.58	13.44	0.67	7.71	5.22	0.27	4.98	0.13
Erysipelotrichaceae	1.77	1.73	0.94	0.26	0.55	0.07	0.78	0.08
Peptostreptococcaceae	1.99	1.57	0.45	3.14	0.94	0.04	0.54	0.02
Ruminococcaceae	0.42	0.34	0.34	1.59	1.46	0.62	2.02	0.28
Enterobacteriaceae	46.12	39.19	0.17	51.19	35.88	<0.001	32.20	<0.001
**Genus**								
Bacteroides	7.48	7.02	0.89	4.54	12.56	0.009	13.92	0.002
Bifidobacterium	11.64	14.73	0.32	9.12	16.83	0.006	16.33	0.006
Clostridium	14.53	13.38	0.67	7.44	5.09	0.27	4.78	0.11
Enterobacter	30.31	24.89	0.21	32.32	18.21	<0.001	15.02	<0.001
Enterococcus	12.40	16.99	0.03	12.72	13.75	0.65	16.12	0.18
Escherichia/Shigella	15.05	13.67	0.73	16.39	15.88	0.73	15.59	0.67
Ruminococcus	0.22	0.17	0.43	0.50	0.56	0.58	1.35	0.01
